# Hepatic Encephalopathy Without Hyperammonemia in the Setting of Liver Metastases by a Pancreatic Neuroendocrine Tumor

**DOI:** 10.7759/cureus.71999

**Published:** 2024-10-21

**Authors:** Natsuki Toyama, Ryousuke Tsukamoto, Makoto Kuroda, Yoshinori Noguchi, Tomoko Sairenji, Yasuhiro Osugi

**Affiliations:** 1 Department of General and Family Medicine, Fujita Health University, Toyoake, JPN; 2 Department of General Practice, Toyota Regional Medical Center, Toyota, JPN; 3 Department of Laboratory, Toyota Regional Medical Center, Toyota, JPN; 4 Department of Family Medicine, University of Washington School of Medicine, Seattle, USA

**Keywords:** altered mental state, altered mental status evaluation, hyperammonemia-encephalopathy, overt hepatic encephalopathy, pancreatic neuroendocrine tumor

## Abstract

Hepatic encephalopathy (HE) is a neurological impairment that typically occurs in patients with liver dysfunction or portosystemic shunting. Diagnosing HE can be challenging since it requires a process of exclusion. Ammonia is considered a major contributor to HE, though ruling out HE solely based on ammonia levels has the potential for misdiagnosis.

Malignancy infiltration is uncommon as an etiology of HE, although there are reported cases of HE patients with pancreatic neuroendocrine tumors (PNETs) diagnosed by the presence of hyperammonemia. We report a case of a disoriented patient with a PNET and diffuse metastases to the liver who presented without hyperammonemia. After excluding possible etiologies of altered mental status, we diagnosed the patient with HE and started on lactulose, which improved his condition. PNET patients can experience HE without hyperammonemia, and a thorough evaluation to rule out other etiologies is necessary for the diagnosis.

## Introduction

Hepatic encephalopathy (HE) is a potentially reversible impairment of neuropsychiatric function with a broad spectrum of symptoms, ranging from subclinical cognitive deficits to coma [1]. HE is mainly observed in patients with acute liver failure, portosystemic shunts, and cirrhosis [1]. Malignant liver infiltration is uncommon as an etiology [2], although several cases of HE due to pancreatic neuroendocrine tumors (PNETs) have been reported.

Diagnosing HE can be challenging since it remains a diagnosis of exclusion [3]. No laboratory values, specific clinical symptoms, or imaging can verify HE. Among causal hypotheses, ammonia is considered a major contributing factor and is measured in daily practice. However, it is known that overt HE patients can present with ammonia levels within the normal range, and past studies report the risk of misdiagnosing HE when judged solely by ammonia levels [4-6]. Additionally, a precise mechanism of HE is yet to be discovered, and other pathogenesis is studied as potential contributors, such as oxidative stress, systemic inflammation, and impaired lactate metabolism [3]. Thus, a thorough examination is required to exclude a wide range of differential diagnoses [1]. 

We experienced a case of suspected HE in the setting of diffuse liver metastasis secondary to PNET, with no elevated serum ammonia. We will also explain how we diagnosed and treated the patient.

## Case presentation

**Figure 1:**
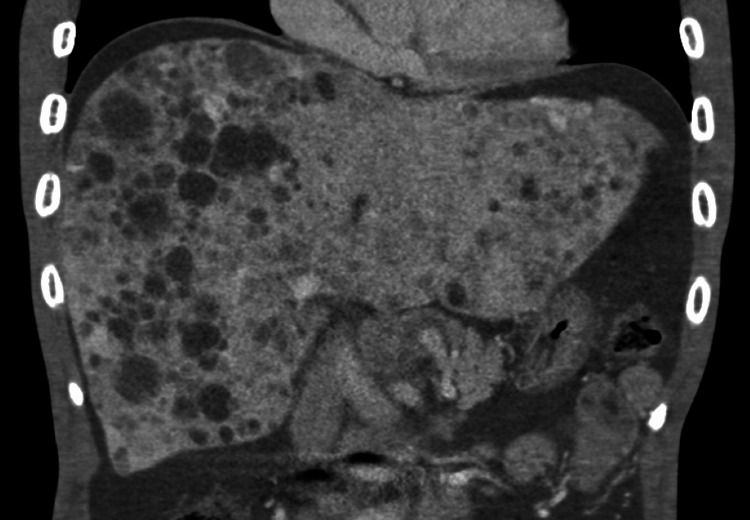
Computed tomography of the abdomen

Our case involves a 58-year-old man without significant past medical history who presented with bilateral edema of the lower extremities, abdominal pain, and nausea, which persisted for a month. Blood tests were positive for elevated liver function and microcytic anemia, and a physical examination was notable for a distended abdomen and palpable epigastric mass. A computed tomography (CT) scan revealed a pancreatic tumor, diffuse liver masses, and ascites. Collaterals and tumor invasion of portal veins were not observed (Figure 1).

**Figure 2:**
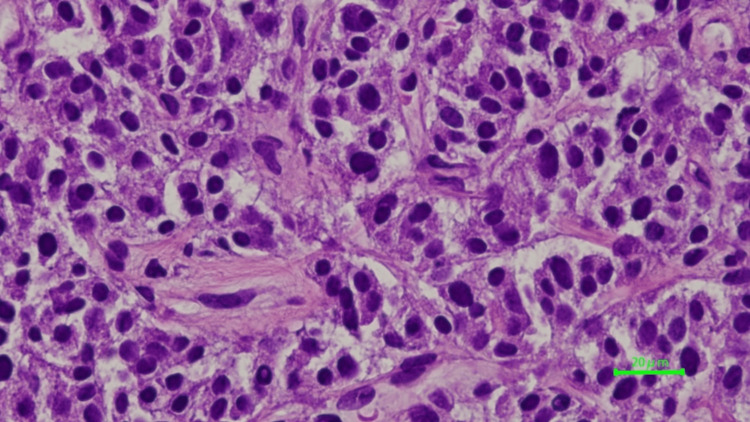
Fine needle aspiration of the liver lesion

A fine needle biopsy of the liver lesion confirmed a non-functional pancreatic neuroendocrine tumor with strong synaptophysin staining, weak CD56 staining, and negative chromogranin A staining. The malignancy grade was determined to be G2, based on an MIB-1 expression rate of 3.0% (Figure 2).

The patient chose a palliative care approach, and he was started on diuretics (daily doses of furosemide 20mg and spironolactone 50mg by mouth) and received paracenteses as needed for comfort.

Three months after the initial diagnosis, the patient visited an emergency department with altered mental status and abdominal pain. He was afebrile, and other vital signs were unremarkable. The Glasgow Coma Scale was 14; he was not oriented to time and slurred speech was observed. Physical examination revealed no signs of infection, and his abdomen was soft and non-tender. He denied melena and rectal bleeding. His neurological examination was positive for asterixis, a newly observed symptom. There were no new medications, and he also denied alcohol or other substances. Psychometric tests were not conducted.

Laboratory work-up was negative for infectious and metabolic causes; his lab work showed largely unremarkable findings, demonstrated in Table 1.

**Table 1:** The result of laboratory workup

Tests	Time of presentation	Three weeks prior	Normal reference range (unit)
White blood cells	7.2	7	4.5-11.0 (x10³/μL)
Hemoglobin	16.2	16.1	14-18 (g/dL)
Platelets	17.5	28.4	15-35 (x10³/μL)
Blood urea nitrogen	38.6	24.9	8-23 (mg/dL)
Creatinine	1.78	1.42	0.6-1.2 (mg/dL)
Aspartate aminotransferase	67	94	10-30 (U/L)
Alanine aminotransferase	53	74	10-40 (U/L)
Alkaline phosphatase	392	475	30-120 (IU/L)
G-glutamyl transpeptidase	220	303	2-30 (IU/L)
Total bilirubin	1.3	1.2	0.3-1.2 (mg/dL)
Ammonia	21	***	9-47 (μmol/L)
Glucose	102	***	
Sodium	138	137	138-146 (mEq/L)
Potassium	5.0	4.7	2.5-4.7 (mEq/L)
Chloride	101	101	99-109 (mEq/L)
Calcium	8.0	***	8.7-10.3 (mEq/L)
Albumin	1.9	2.6	4.0-5.0 (g/dL)
PT-INR	0.94	***	0.85-1.15
ChE	162	***	214-466 (U/L)

A head CT scan was unremarkable for intracranial pathology, and the abdominal CT scan did not reveal new findings. Due to worsened kidney function, diuretics were discontinued. The patient initially complained of abdominal pain; however, the pain gradually subsided, and no analgesics were administered. Similarly, no psychotropic medications that could affect mental function were given. 

The patient’s mental status was unchanged for the next three days. Examinations and tests did not provide other possible etiologies of disorientation, such as hypoglycemia, liver failure, sepsis, electrolyte abnormalities, and intracranial hemorrhage and metastasis. 

By excluding other causes of altered mental status, he was suspected of having HE and was started on oral 65% lactulose syrup (90ml) and branched-chain amino acids (150mg) daily. Three days after initiating the medications, his mentation cleared to his initial level of Glasgow Coma Scale of 15, and asterixis was no longer observed.

He was maintained on these medications, and his mental status remained clear for the following three weeks, and he died from respiratory failure four weeks later.

## Discussion

Diagnosing HE requires detecting signs suggestive of HE and excluding disorders that cause altered mental status [1]. A cognitive function test is performed to support the diagnosis [1]. Elevated ammonia levels raise suspicion of HE. However, that alone does not add diagnostic value in chronic liver disease patients [1]. This is because serum ammonia levels can vary based on their underlying liver function, sarcopenia, diet renal function, and handling of the samples [6].

Even if ammonia levels are within the normal range, ruling out a diagnosis of HE only based on ammonia levels has the potential for misdiagnosis. It is known that overt HE patients can present with ammonia levels within the normal range [4-6]. Haj and Rockey reported that 40% of the patients with cirrhosis who presented with altered mental status had normal ammonia levels [6]. In addition, a precise mechanism of HE is yet to be discovered, and other potential contributors, such as oxidative stress, systemic inflammation, and impaired lactate metabolism, are being studied [3]. 

Our patient had a PNET with diffuse liver metastases and presented with acute altered mental status, and hyperammonemia was not present. Malignancy infiltration is an uncommon etiology of HE [6], although several cases of HE with underlying PNETs have been reported. All the cases accompanied elevated ammonia levels (Table 2).

**Table 2:** Reported cases of hepatic encephalopathy with underlying pancreatic neuroendocrine tumors

Reference	Case	Serum ammonia level	Possible underlying cause	Treatment
Al-Bawardy et al., 2013 [7]	56-year-old male	174μmol/L	Microvascular portosystemic shunting	Lactulose ineffective
Monardo et al., 2020 [8]	47-year-old male	163μmol/L	Hormones and neurotransmitters, dehydration	Distal pancreatectomy
Broadbridge et al., 2010 [9]	78-year-old male	151μmol/L	Portal hypertension	Lactulose, dialysis
Broadbridge et al., 2010 [9]	52-year-old male	88μmol/L	Pneumonia, minor progression of disease	Antibiotics, lactulose
Zorgdrager et al., 2023 [10]	36-year-old male	185μmol/L	Portosystemic shunts	Lactulose
Erinjeri et al., 2009 [11]	63-year-old male	131μmol/L	Hepatofugal flow	Hepatic artery embolization
Pande et al., 2016 [12]	45-year-old male	Greater than 500 μmol/L	Portosystemic shunts	Transarterial chemoembolization
Davis et al., 2021 [13]	55-year-old female	170μmol/L	Portosystemic shunts	Dexamethasone, transarterial chemoembolization

We diagnosed our patient with HE based on the following clinical reasoning.

The physical examinations, laboratory tests, and imaging did not provide other possible etiologies of altered mental status. Our patient had no intracranial pathology such as hemorrhage and mass, electrolyte disorders, infections, or end-organ failure, which are the major etiologies of cancer-related confusion [2]. There was no history of consumption of recreational drugs, alcohol, or new medications. Additionally, though non-specific, we observed new bilateral asterixis at the initial presentation. This is usually due to metabolic encephalopathies including HE, respiratory failure, azotemia, and other etiologies [14]. The physical examinations and tests were unlikely for these etiologies as well, and his underlying disease made HE the most likely cause of his altered mental status.

In our case, dehydration is a possible precipitating factor of HE since there was an increase in blood urea nitrogen and urine creatinine ratio, which could have resulted from diuretics the patient was taking regularly. We cannot provide a definitive reason for the lack of hyperammonemia. It is possible that pathogenesis, such as lactate levels in the brain and neurotransmitter dysfunction, which are the potential pathogenesis of HE, were related to the development of HE in the patient [3]. 

Another reason to think that HE is suggestive in this case was the patient’s rapid improvement after initiating the treatment for HE. Initiation of lactulose and BCAA were the only interventions made, but the patient’s mental status improved within three days and remained stable until his overall condition worsened. Oral lactulose improves cognitive functions and health-related quality of life even in minimal HE patients without prominent hyperammonemia [15]. Recent studies suggest that lactulose affects not only gut microbiota but also influences the conjugation of bile acids, which may be another reason for lactulose being effective since elevated cranial levels of bile acids have been reported in HE patients [3].

Our case highlights the importance of not omitting HE from differential diagnosis with the serum ammonia level alone in PNET patients. Based on our literature search on PubMed, conducted in January 2024, using “neuroendocrine tumor AND hepatic encephalopathy” as a keyword, elevated ammonia levels accompanied all the cases. 

## Conclusions

In conclusion, it is inducible that our patient presented with HE, and we were able to reverse symptoms by initiating appropriate treatment. We believe that it is essential to note that PNET patients can also experience HE without hyperammonemia, and a thorough evaluation to rule out other possible diagnoses should be done to make the diagnosis.
